# The 5G COVID-19 Digital Wildfire: An evolving network of Twitter contacts to explore phase transition metaphors in viral misinformation

**DOI:** 10.1371/journal.pone.0343661

**Published:** 2026-03-13

**Authors:** Kaspara Skovli Gåsvær, Pedro G. Lind, Johannes Langguth, Morten Hjorth-Jensen, Michael Kreil, Daniel Thilo Schroeder

**Affiliations:** 1 Department of Physics and Center for Computing in Science Education, University of Oslo, Oslo, Norway; 2 School of Economics, Innovation and Technology, Kristiania University of Applied Sciences, Oslo, Norway; 3 Department of Computer Science, OsloMet–Oslo Metropolitan University, Oslo, Norway; 4 Department for High Performance Computing, Simula Research Laboratory, Oslo, Norway; 5 Bayerischer Rundfunk, Munich, Germany; 6 Department for Sustainable Communication Technologies, SINTEF, Oslo, Norway; Bogazici University, TÜRKIYE

## Abstract

Shortly after the first COVID-19 cases became apparent in December 2019, rumors spread on social media suggesting a connection between the virus and the 5G radiation emanating from the recently deployed telecommunications network. In the course of the following weeks, this idea gained increasing popularity, and various alleged explanations for how such a connection manifests emerged. Ultimately, after being amplified by prominent conspiracy theorists, a series of arson attacks on telecommunication equipment followed, concluding with the kidnapping of telecommunication technicians in Peru. In this paper, we study the spread of content related to a conspiracy theory with harmful consequences, a so-called Digital Wildfire (DW). In particular, we investigate the 5G and COVID-19 DW on Twitter before, during, and after its peak in April and May 2020. For this purpose, we examine the community dynamics in complex temporal interaction networks underlying Twitter user activity. We assess the evolution of this particular DW by appropriately defining the temporal dynamics of communication in communities within social networks. We observe that, for this specific DW, the number of interactions of the users participating in the DW, as well as the size of the engaged communities, both exhibit visual patterns suggestive of power-law distributions, consistent with other social networks, though based on exploratory inspection rather than formal statistical tests. Moreover, our exploratory analysis elucidates the possibility of conceptualizing the phases of a DW, as per established literature. We identify one such phase as a potential critical shift, marked by a shift from sporadic tweets to a global spreading event, highlighting patterns suggestive of dramatic scaling in misinformation propagation, used heuristically without quantitative physical parameters. Additionally, we argue that patterns suggest the observed shift is associated with influential users, who appear to amplify the spread of misinformation, though causality is exploratory. Lastly, our data suggest that the characteristics of such events could contribute to prediction models, at least in some instances. From this data, we hypothesize that monitoring minor peaks in user interactions, which precede the critical phase culminating in real-world consequences, could serve as an early warning system, aiding in the anticipation and potentially the mitigation of DWs.

## Introduction and background

Before the advent of the Internet, people primarily relied on print media, TV, and radio as their main sources of news. During that era, information dissemination was characterized by a clear separation between the source and the consumer. The flow of information was unidirectional and slow-paced, and it was common practice to trust journalists and newspapers as authoritative and reliable sources of information.

The inception of the Internet [1] on January 1, 1983, marked a turning point in the way we exchange and receive information. Initially, the Internet was primarily populated by users with expertise in science, technology, engineering, and mathematics. At this stage, discussions were restricted to a small audience, and traditional broadcasting and media outlets still held sway. However, a significant shift occurred in the late 1990s with the introduction of the first online social networks (OSNs) like Bolt.com [2] or SixDegrees.com [3], which opened the gates for diverse individuals to participate in a broader online discourse. With the ensuing absence of a clear separation between information sources and consumers, the issue of the trustworthiness of the sources started to become more pressing.

The reliability of news agencies today can vary widely depending on the country and agency in question. Nonetheless, the mechanisms for accountability tend to be more formalized for news agencies than for OSNs. While fact-checking of social media content may occur in rare cases, it typically only happens after posts reach a large audience [4], and even then, the desired effect often fails to materialize [5]. Despite these efforts, a remaining challenge for fact-checking is the extreme amount of data available online. Although estimates for the amount of produced data vary depending on the source and the definition of ’data’, it is estimated that the amount of data produced globally has been growing exponentially in recent years. According to a report by Seagate and IDC from 2020 [6], the global datasphere – which includes all the data created, captured, and replicated in a year – was projected to reach 175 zettabytes by 2025. Even though OSN data is only a small part of this, its sheer volume and velocity overwhelm current monitoring approaches, and we cannot yet reliably detect misinformation with viral potential before it escalates. Thus, misinformation often spreads unnoticed, especially on social media. We argue that the fact that (1) anyone, regardless of their qualifications, can post about anything online, (2) the resulting sheer amount of unchecked misinformation, and (3) the lack of accountability imposed on the providers of OSNs, turns the sea of online information into a maze; tricky to navigate even if one is aware of these challenges, and potentially dangerous if not.

As a result, misinformation travels at a speed never previously seen, possibly resulting in severe real-world implications [7–11]. These phenomena are called Digital Wildfires (DWs) [12] and defined as the rapid spread of information or rumors, amplified by the power of social media, which can create significant societal and economic damage in a short amount of time. The dynamics of such rapid spreading events have theoretical roots in information cascade models, where local interactions can trigger global propagation, a concept explored in early network science literature [13]. The term DW we use in this paper extends this definition by adding a temporal dimension.

As a result, misinformation can travel at speeds higher than real information, possibly resulting in severe real-world implications [7–11]. These phenomena are called Digital Wildfires (DWs) [12] and are defined as events with rapid spread of information or rumors, amplified by the power of social media, which can create significant societal and economic damage in a short amount of time. The dynamics of such rapid spreading events have theoretical roots in information cascade models, where local interactions can trigger global propagation phenomena, generating collective macroscopic behavior as observed in classical models such as percolation model to describe fluid permeability in porous medium, or the Ising-model to describe ferromagnetic phenomena. This conceptual connection between phase transitions in statistical physics and information cascades in social/complex systems as been widely reported and exemplified -cf. Ref. [13,14].

In the context of DWs, the globally spread state is triggered by the first social media post that addresses the potentially fast-spreading topic and end after real-world consequences occur. Langguth et al. [15] have shown that the topic leading to real-world consequences may continue to be discussed even after these consequences occur. Furthermore, a new DW may emerge around the same complex of topics in a different context. However, for this article, we refrain from such an extended definition and stick to defining the lifecycle of a DW from the first social media post until after the real-world consequences have occurred.

To effectively combat the rise of DWs, it is vital to establish automated systems capable of early misinformation detection, thereby allowing for prompt interventions. A large body of research has studied the automated detection of misinformation using approaches such as linguistic-based [16], visual-based [17], user-based [18], post-based [19,20], and network-based detection [21,22]. Despite these efforts, a comprehensive strategy targeting DWs specifically remains undeveloped.

Thus, before devising effective automated systems, it is imperative to delve deeper into understanding the mechanisms and dynamics fostering the proliferation of DWs. Acquiring such knowledge lays a crucial foundation for crafting robust and precise algorithms vital for early detection and prevention.

In this article, we aim for a generic approach exploiting not only the content but rather the underlying interactions of a particular DW, namely *the 5G and COVID-19 misinformation event* [15], within the OSN Twitter to gain knowledge about the properties and dynamics of the spread of DWs on a societal scale. Specifically, we investigate the evolution of the temporal networks induced by the interactions between Twitter users to uncover the emergence of DW.

**Fig 1:**
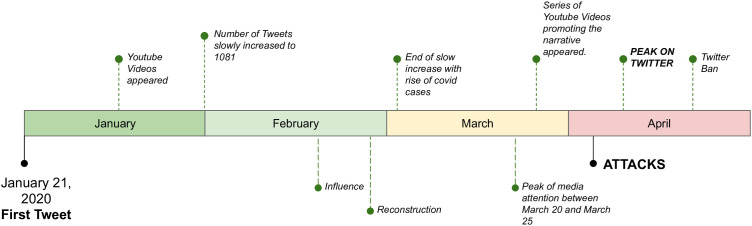
Timeline of the most significant events that occurred during the course of the COVID-19 and 5G DW in 2020.

Previous research has shown that investigating only the diffusion pattern of this kind of misinformation on an individual, per social media post basis is not promising at all [21,23]. Even more, it seems like we can only understand a DW when examining the entirety of information cascades associated with it [24]. In this paper, we address the following question: Given the interaction data of an entire DW from the online social network Twitter, can we explain its dynamics and temporal evolution on a societal scale by using complex and temporal networks? The specific temporal network we study originates from interactions between Twitter users connected to the 5G and COVID-19 DW, a series of tweets claiming a link between the COVID-19 virus and 5G technology that lead to a DW. This DW reached its peak around April *2020* (see Fig 1), resulting, among other things, in the destruction of 5G-related telecommunication equipment and the harassment as well as the kidnapping of telecommunication workers.

We undertake an examination of the temporal evolution of interaction networks — precisely focusing on the dissemination of misinformation surrounding the 5G and COVID-19 event as it unfolded on Twitter. Individual information cascades on this platform predominantly manifest through threads of tweets and retweets. Understanding that DWs are essentially conglomerates of numerous such cascades, we venture to scrutinize the evolution of the DW through a lens encompassing a significantly comprehensive set of related cascades — a holistic approach facilitated by our comprehensive dataset that, to the best of our knowledge, stands as the most expansive in network-based study of DWs. Using community detection methods [25–27], we delve into discerning the dynamics. Furthermore, by evaluating both the centrality and activity of the vertices, we aim to pinpoint the roles and repercussions of group and individual activities in steering the temporal trajectory of the network.

Our objective is to investigate dynamics to describe and predict the evolution of complex temporal networks related to the spread of DWs. The insights gained from this study can aid in the early detection and prevention of misinformation events before they escalate into DWs and end with real-world consequences.

In the following, we list our contributions to the understanding of the 5G and Covid-19 DW. First, we observe that the DW displays patterns suggestive of phase transition behavior, highlighting the need to approach this phenomenon from a complex systems and network science perspective. Second, our investigation of community dynamics reveals a synchronization of communities towards the peak of the DW, which corresponds to the time when real-world consequences occur. Third, we identify a small group of influential users who appear associated with amplifying the conversation on a large scale, drawing in a significant number of new users, though causality is exploratory and could be tested via simulations in future work. Finally, our analysis of the largest cluster shows that it is unstable and characterized by oscillations of partly contradictory narratives.

Previous work addresses similar questions and problems. Vosoughi et al. [24] investigate the dissemination of true and false news on Twitter, as detailed in their 2018 study. The authors analyze a dataset encompassing approximately 126,000 news stories tweeted by around three million users, assembled into rumor cascades. Their findings underscore that false news stories propagate significantly faster, reach deeper, and spread more broadly than true stories. Furthermore, false information was found to be retweeted more frequently than true news. Intriguingly, their findings indicate that true stories take approximately six times longer to reach a similar audience size of 1,500 users compared to their false counterparts.

Starbird [28] analyzes Twitter data related to eight mass shooting events from 2013 to 2016, identifying alternative narratives that emerged on the platform. These alternative narratives often contradict mainstream media reports and suggest that the shootings were false flag operations or hoaxes. The study uncovers that alternative media sources play a central role in the production and dissemination of these narratives, acting as key amplifiers of misinformation. The research also highlights the interconnected nature of the alternative media ecosystem, with multiple alternative media sources cross-promoting each other’s content and reinforcing the alternative narratives. This interconnectedness contributes to the spread of misinformation and fosters distrust in mainstream media sources.

Del Vicario et al. [29] conducted a study investigating the spread of misinformation on social media, with a specific focus on Facebook. Their research identifies similar consumption patterns among users who prefer scientific news and users who prefer conspiracy theories. However, the patterns of information spreading, or *cascade dynamics*, shows differences. The authors discover that users tend to form *echo chambers*, polarized, homogenous clusters where they share content that aligns with their beliefs. Furthermore, the authors introduce a data-driven model that successfully mimicked these dynamics, reinforcing that homogeneity and polarization are key determinants of content spread.

In their study, Friggeri et al. [30] examine the dynamics of rumor propagation on Facebook. They find that rumors, irrespective of their veracity, spread deeply through social networks, with true rumors generating larger cascades. Their propagation continues even after debunking, indicating that users might overlook or ignore debunking comments. Furthermore, they observe that the popularity of rumors is bursty, with humor sometimes serving as an antidote to rumor propagation. However, despite these findings, the authors acknowledge potential biases in their sample collection and analysis.

Langguth et al.’s study [15] expanded on the concept of DWs, focusing on the event we aim to research in this study, the misinformation linking 5G technology with the COVID-19 pandemic. They trace the origin of this rumor and reveal how it grew across social media platforms. The study encounters that even contradictory narratives could strengthen DWs, and that the role of commercially-influenced videos is often underestimated in Twitter-only analyses. The authors suggest several countermeasures, including focusing on the financial motivations behind the spread of misinformation in general and DWs in particular and promoting international cooperation in research on DWs. However, the analyses in this study are more qualitative in nature and do not include a structural analysis of the underlying communication in networks.

### COVID-19 and 5G conspiracy theories as a specific Digital Wildfire case

As the COVID-19 pandemic swept across the globe in early 2020, a proliferation of tweets occurred linking the virus’s origins to 5G wireless technology. Initially confined to a small and insignificant number, the volume of such tweets surged exponentially throughout April 2020, culminating in a series of arson attacks on 5G towers in multiple countries, including the United Kingdom [31], Nigeria [32], and Canada [33]. As mentioned in the previous section, formally, such fast-growing dissemination of online misinformation leading to real-world implications is known as a Digital Wildfire (DW) and ranked as a top global risk by the World Economic Forum [12].

In the following, we introduce the chronology of the 5G and COVID-19 DW delineated into three distinct phases, a classification grounded in a qualitative evaluation of the DW: pre-real world events, during-real world events, and post-real world events. The demarcation of these phases serves as a framework for unraveling the intricate dynamics at play. Notably, the event persists to this day, warranting continued scrutiny. However, this study is confined to exploring the events until May 2020. For a more detailed overview of the DW under investigation, including developments stretching to late 2022, we refer to the assessment provided by Langguth et al. [15].

### Pre-real world event

With the first tweet collected in early January, we observed a slow growth in daily tweets, insinuating a connection between COVID-19 and 5G throughout January and February 2020. In addition, we note the gradual uptick in interest for such content on platforms beyond Twitter, including notable activity on YouTube. Pinpointing the exact inception point, however, presents a challenge due to the presence of multiple sub-narratives that are arguably leading to the DW. Furthermore, we use Twitter data only, leaving open the possibility that discussions regarding the event were initiated on a platform other than Twitter. However, when investigating the early tweets, we discover an entire spectrum of conspiracy narratives claiming a causality between 5G radiation and the coronavirus. Even though these narratives seem to be as diverse as the individuals spreading them, they share the idea that the 5G technology is dangerous, can hurt people, and thus should not be implemented. For detailed descriptions and tweet samples for subnarratives, we point to the datasets published by Pogorelov et al. [34] and Schroeder et al. [23]. At this point, we would like to point out that before the end of January 2020, only 685 tweets and 1,081 retweets containing both keywords referencing COVID-19 and 5G appeared. This comparatively small number has led to the decision to start the period of observation on the first of February in this study.

### During-real world event

In March, when the pandemic began to gain a foothold in Europe, the daily number of tweets quadrupled from late March to early April. Subsequently, the first series of arson attacks happened in the UK, the Netherlands, and New Zealand during the weekend of April 3, 2020. Multiple more followed in the week after, and later some occurred in Canada as well. By July 2, 2020, there were reports of 273 cases of clashes between people who believed in some version of the conspiracy theory, 121 reports of arson and other types of destruction of property [35], as well as the detainment of 8 telecommunication workers in Peru.

### Post-real world event

In late April of 2020, Twitter banned material and users promoting attacks on 5G infrastructure, and the spreading of content related to the connection seemed to halt. However, even as late as the first quarter of 2021, suspected cases of arson in Africa and Canada [36,37] started to occur.

In this paper, we recognize the phase characterized as “during-real world events” as indicative of a phase transition phenomenon, a concept borrowed from the field of statistical physics [14,38]. This classification draws parallels with transitions seen in critical phenomena such as percolation, a well-studied concept in physics. As we delve deeper, it will be evident that the evolution of the DW during the COVID-19 and 5G DW exhibits characteristics akin to the onset of a percolation threshold, signifying a critical phase in the information dissemination process.

### Data collection and preprocessing of massive Twitter datasets

Since Twitter‘s Terms of Service, at the time, prohibited storing large datasets, we choose a streaming-based approach which was first introduced in Schroeder et al. [39]. We keep only in-stream-anonymized user IDs, the corresponding timestamps, and texts to create the tweet-retweet-user mapping. Moreover, we neither store nor process any other information. The data collection took place using a custom build framework for Twitter graph analysis similar to the one presented by Schroeder et al. [40] and a custom scraping strategy similar to the one presented by Burchard et al. [41].

Since Twitter’s search API, at that time, only returned tweets that were not older than two weeks, it was necessary to collect the streamed data preemptively while hoping that this collection then, at the time of a DW, would contain the relevant tweets. In the following, we describe exactly this procedure.

Between December 2019 and May 2020, we collected a total of 6,286,886,977 COVID-19-related tweets, retweets, replies, and quotes (referred to as statuses) leveraging the keywords outlined below and using Twitter’s search API. It is pertinent to note that querying the Twitter search API frequently yields duplicate entries. To ensure the robustness of our dataset, we meticulously identified and removed these duplicates in the initial phase of our data processing. This filtration process resulted in a refined dataset comprising 2,570,581,178 unique statuses, which formed the basis for our subsequent analysis. The keywords for initial Twitter API search were: *coronavirus, corinavirus, corona, coronaoutbreak, coronavirus, coronavirusde, coronavirusoutbreak, covid, covid19, covid2019, covid_19, covid-19, wuhancoronavirus, wuhanvirus, coronavírus, coronavirus, coronavirus8, coronavirus, zerocovid, coronar_allesoeffnen, allesoeffnen, allesöffnen, coronadeutchland, xj621, machtbueroszu, machtdiebueroszu, bueroszu, büroszu, diebüroszu, vaccination, vaccine, epidemic, pandemic, quarantine, quarantined, mutation, wuhan, coronapanik, covidiot*.

Next, we filtered for tweets that mention " 5G " and " 5g “, avoiding whitespace removal to reduce false positives unrelated to 5G, and include alternative spellings like " 5-G “, although these are negligible. After applying this filter and excluding statuses outside February 1, 2020, to May 11, 2020, 364,325 COVID-19- and 5G-related tweets remain, spanning 100 days. This deliberate choice prioritizes depth over breadth, as it is common in exploratory social media studies [24,28]. However, it may miss subtler sub-narratives (e.g., indirect references like “radiation causing the virus” or slang like “fifth gen towers”), potentially underestimating early DW percolation or cross-platform influences (e.g., YouTube videos [15]). The English-heavy keyword list may underrepresent non-English narratives, limiting insights into global dynamics given real-world impacts in multiple countries. Additionally, coincidental co-mentions (e.g., telecom ads using “5G”) were rare in our broader 5G-COVID-19 related work [34].

The enrichment phase commenced with the curated dataset derived from the preceding filtering phase. Central to this phase is the process of Twitter thread completion. To elucidate, a Twitter thread is a cohesive series of interconnected tweets stemming from an inaugural tweet and encapsulating all ensuing replies and quoted tweets to foster a consolidated conversation. Such threads offer a structured vantage point, enabling a comprehensive insight into the contextual dynamics enveloping the primary tweet. During this phase, individual threads pertaining to a specified tweet are queried to facilitate the incorporation of statuses surpassing the two-week retrieval constraint imposed by the Twitter search API. Consequently, this method permits the inclusion of tweets devoid of any keywords. It warrants mention that despite endeavors to augment the dataset by resurrecting more dialogues linked to the DW, the potential for incomplete threads persists, attributed to the inherent limitations of the Twitter API, which confines queries to parent statuses within a thread exclusively. Notwithstanding this limitation, we note that the augmentation process, albeit yielding incomplete threads, substantially enhances the dataset by infusing it with valuable context.

Following the enrichment phase, we engaged in a detailed analysis of the resulting dataset, a process that fostered the foundation for multiple scientific publications. One significant step in this process was the meticulous labeling of over 9,688 tweets to determine their association with the 5G COVID-19 DW. This effort yielded two distinct datasets: one archiving the tweets and another detailing 3,492 individual tweet-retweet cascades, each labeled to indicate association with the DW. These datasets, denominated as WICO-Text and WICO-Graph, are discussed in depth in works by Pogorelov et al. [34] and Schroeder et al. [23], respectively. To further leverage these datasets, we organised a MediaEval Benchmark Challenge task, wherein both datasets were subdivided into testing and training sets, and distributed to an initial pool of 15 groups. These groups embarked on developing distinct classifiers capable of differentiating between tweets and cascades genuinely associated with the DW. The entire endeavor is documented in detail in Pogorelov et al. [21].

The described data collection, anonymization, storage, and use have been reviewed by the Ethical Committee of the Simula Research Laboratory in Oslo and are in accordance with the relevant guidelines and regulations. We see no ethical concerns with the data use or the publication of results based on the data. Furthermore, the Ethics Committee confirmed that no participant consent was required and formally waived the need for informed consent.

Concerning data availability, in accordance with Twitter’s Terms of Service and to ensure user privacy, we will not publish or share the underlying Twitter source data collected during our study. Our methodology involved the storage of in-stream-anonymized user IDs, timestamps, and the texts necessary to form the tweet-retweet-user mapping, strictly omitting any other personal information. However, we have created interaction networks derived from the processed Twitter data, which elucidates the structural and relational aspects that informed our analysis, while being compliant with privacy and ethical guidelines. These interaction networks, which formed the foundation of our research, encapsulate the connectivity and dynamics pertaining to the specific period of study from February 1, 2020, to May 10, 2020 and are available under https://osf.io/vqhet/?view_only=6b67055f78e047349036332f5bab7365. The corresponding codebase is available under https://github.com/KasparaGaasvaer/MasterThesis.

### From temporal interactions to interaction networks

Given the filtered and enriched dataset, we now extract user interaction by counting contacts between each pair of users. We define $Z_{u}=\text{set of users }$ and $Z_{s}=\text{set of statuses }$. A contact between two users is defined as


any user j interacting with any user i through user j either retweeting, replying, or quoting user i.  (1)

The set of contacts induces a symmetric adjacency matrix *A* with $A_{ij}=1\;$ to label an existing contact between users *i* and *j* and *0* if such contact does not exist. By keeping track of the number of retweets, replies, and quotes between users, we are able to build the directed and weighted network of interactions. Since in this paper, we focus on assessing the size of connected users, we consider, for simplicity, the contacts as unweighted and undirected edges, forming the interaction network and its communities. Furthermore, we call such a network temporal interaction network when contacts have timestamps allowing for only considering excerpts of an interaction network within an arbitrary time window. More precisely, we build temporal interaction networks based on the adjacency matrix *A*, defining the *underlying graph* $G_{↓}=(V_{↓},E_{↓},t$ as the temporal graph containing the entirety of vertices and edges where $V_{↓}\;$ is the set of vertices representing the users, $E_{↓}\subseteq V_{↓}\times V_{↓}\;$ is the set of all edges, and $t:E_{↓}\to [0,T]\;$ is the timestep function which assigns a timestep to each edge. *T* is the length of the window of observation. Timesteps are aligned with the start of the window of observation and are thus nonnegative, and *T* is also the time at the end of the observation.

### Assessing the evolution of interaction networks

To examine the network dynamics in a temporal way, we divide $G_{↓}\;$ into slices of even length Δt . Let L=T/Δt  be an integer.

**Fig 2:**
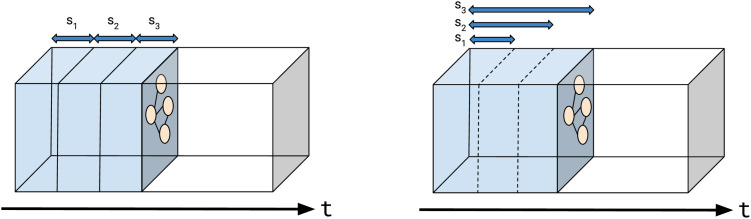
(Left) Illustration of the concept of temporal slices and (Right) accumulative slices. Temporal slices do not contain the vertices and edges of previous slices, while accumulative slices contain all vertices and edges from the previous slices.

Although the underlying graph is temporal, each slice is a static “snap-shot” of a time period in the interaction network. In the following, we present two distinct types of slices: temporal slices and accumulative slices (see Fig 2). The rationale behind developing multiple slice types is the potential to extract diverse information from each one.

Thus, we obtain temporal slices $G_{i}=(V_{↓},E_{i}),1\leq i\leq L\;$ where $E_{i}=\{e|e\in E_{↓}\wedge (i-1Deltat\leq t(e)\leq i\Delta t\}\;$. Accumulative slices are simply the union of a temporal slice with all preceding temporal slices. Thus $S_{i}=(V_{↓},E_{0}\cup E_{1}\cup \ldots \cup E_{i}$.

In the context of network analysis, pure temporal slices offer valuable insights into the overall temporal evolution of the system. However, their limitation lies in their inability to trace clusters across multiple slices because of the removal of nonactive vertices in subsequent time periods. Alternatively, accumulative slices provide the advantage of cluster tracking but come with the disadvantage of rapidly increasing size, posing a challenge in handling them effectively.

We divide the interaction network into slices of sub-graphs based only on the timestamps, and in a non-accumulative manner. Moreover, we remind the reader that edges are contacts, e.g., retweets or comments, and thus associated with a timestamp. We call the duration of a temporal slice Δt  an interval and we chose Δt=24  hours to reflect practical Twitter rhythms capturing daily user activity cycles, and Δt=4  hours for visualizations that reveal finer temporal details in plots, such as in Fig 4. While robustness to alternative Δt  values was explored initially, we present results descriptively to highlight temporal patterns rather than claim statistical invariance, consistent with our exploratory focus.

**Fig 3:**
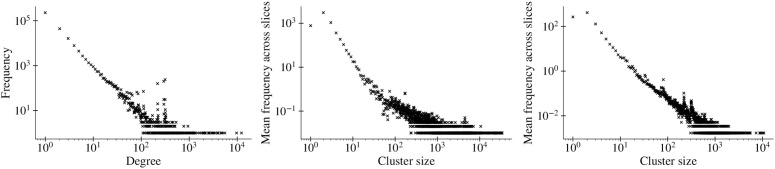
Three-part depiction of Network G_↓_. The left subfigure presents the global degree distribution, illustrating the range of degree centralities. The central subfigure displays the distribution of cluster sizes across all accumulative slices, with clusters identified via the Leiden algorithm [27]. The rightmost subfigure explores the cluster size distribution across all temporal slices (Δt=4h , chosen for visualization to reveal finer temporal details, with Δt=24h  reflecting daily Twitter rhythms). Both the middle and rightmost figures are created by counting the occurrence of clusters with size *C* in every slice before averaging the number of occurrences by the number of slices in the experiment. Notably, both degree and cluster sizes exhibit visual patterns suggestive of power-law distributions, mirroring those found in other social networks. Note that this assertion is based on exploratory inspection rather than formal statistical tests.

### Degree centrality as a proxy for user activity

Centrality measures give insight into which users or vertices contribute the most to the flow of information. As we are dealing with an undirected network, we cannot determine whether a vertex is highly active, e.g., comments on other statuses with a high frequency or is made highly active by others, e.g., many other statuses are responses to a status. Thus, we define vertex activity as *the number of contacts a user experiences*. In other words, this is the number of edges incident to a given vertex. Degree centrality can be calculated for each slice or the entire network G↓ . For temporal slice *i* it is defined as


$${𝒞}_{\text{deg}}^{i}(v)=|{𝒩}^{i}(v)|\;$$ (2)

where ${𝒩}^{i}(v$ is the neighborhood of the vertex *v* in slice *i* defined as


$${𝒩}^{i}(v)=\{u\subset V_{↓}|\{v,u\}\in E_{i}\}\;$$ (3)

Degree centrality in an agglomerative slide $S_{i}\;$ is simply ${\bar{C}}_{\text{deg}}^{i}(v)=\sum_{1\leq k\leq i}{𝒞}_{\text{deg}}^{i}(v$ with ${\bar{C}}_{\text{deg}}^{L}(v)={𝒞}_{\text{deg}↓}(v$ being equal to the degree centrality of *v* in the underlying graph.

Later, we use this measure to explore the correlation between the overall vertex activity of a group and other properties of the system, such as the size of the largest clusters and the number of overall contacts.

Degree centrality was selected for its simplicity and interpretability in illustrating basic diffusion potential in the DW. While alternative metrics such as eigenvector or betweenness centrality may capture different aspects of influence (e.g., embeddedness in highly connected groups or bridging roles), exploring these systematically is beyond the scope of this paper.

### Scaling patterns in user contacts and community sizes

Many empirical studies have shown that social networks exhibit power-law degree- and community-size distributions. The phenomenon occurs across complex networks ranging from social [42,43] to informational [44,45] and biological networks [46,47]. Rigorous statistical methods for identifying such distributions are described by Clauset, Shalizi, and Newman [48], though in this paper we restrict ourselves to exploratory inspection. Here, power law distributions arise for various reasons, such as preferential attachment, i.e., new nodes are more likely to connect to already well-connected nodes; growth, i.e., networks expand over time; and homophily, i.e., similar nodes tend to connect with each other.

For the Internet itself [42] as well as for social networks [49], including Twitter [39], it has been shown that both connectivity in general and the number of communication contacts in particular follow power-law distributions. Fig 3 shows that this also applies to communication within the DW under investigation. As with general communication in social networks, there are many users with few contacts to others as well as few users with many contacts to others. Fig 3 depicts $G_{↓}\;$’s global degree distribution, illustrating the range of degree centralities, the community sizes across all accumulative slices, and the community size distribution across all temporal slices. For this, we investigate the community structure underlying our interaction network using the Leiden algorithm [27] with standard configurations for the resolution parameter. The Leiden method was chosen because it improves upon Louvain by guaranteeing well-connected and stable communities while remaining computationally efficient for large graphs. In our context, these advantages were crucial given the size and temporal slicing of the dataset. Conceptually, clusters represent evolving sub-narratives or conversation arenas in the DW: smaller clusters often reflect tightly knit groups with high internal interaction, whereas larger clusters capture broader but sometimes looser discussions. This distinction helps us interpret whether misinformation spreads primarily through dense, cohesive groups or more diffuse communities.

The power-law degree distributions denote the existence of few hubs, i.e., Twitter users with numerous connections or interactions. Simultaneously, numerous nodes exhibit fewer connections. In the context of DWs, this distribution suggests that these hubs play a critical role in driving the dynamics of interactions within the DW. A small number of users can significantly impact the course of interactions, substantiating their role in the progression of DWs.

Community size distributions following a power law indicate a composition of multiple small communities alongside a few large ones within the DW. While clusters of users exhibit intensive interaction amongst themselves, the network’s major interaction activity concentrates within a handful of large communities, for which we later show that conversation within these communities is mostly driven by influential users.

Power law distributions, in degree and community size, bring forth numerous implications for DWs. The resilience to random node failures associated with power-law degree distributions implies that deactivating or removing a random user might not disrupt the spread of the DW. The highly connected hubs maintain the continuum of interaction. Furthermore, we argue that hubs and large communities significantly influence the conversation direction, narrative shape, and information spread within the DW while network structure facilitates the rapid and broad diffusion of information, ideas, or behaviors, especially if instigated or promoted by the hubs or large communities.

### Defining the life cycle via phase transition

A phase transition is a well-established concept in physics [14,38], with examples such as the transition from a solid to liquid state (melting), from liquid to gaseous (evaporation), or the transition from a set of small disconnected groups of individual to a large set of individuals, spanning an entire society. This latter phase transition is usually called transition to percolation [14], and occurs often within the realm of complex systems and network science, usually associated with the emergence of new structures, functionalities, or patterns. Classic examples of phase transitions in complex systems are the emergence of a giant connected component in a random network [50], or the abrupt transition from free-flowing traffic to a traffic jam, a scenario often referred to as a ’phantom traffic jam’ [51]. Moreover, phase transitions also serve as effective metaphors for sudden changes in collective human behaviors, particularly in the digital realm [52]. A specific idea or movement might transition from being recognized by a limited number of individuals to achieving widespread recognition or even reaching viral status.

**Fig 4:**
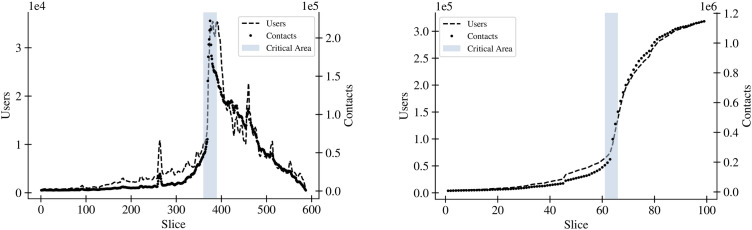
(Left) Total number of users and established contacts in each time “slice” and (Right) the corresponding accumulative slices. Contacts encompass retweets, quotes, and comments, along with the count of distinct users per time segment. The 100-day investigation span, from February 1, 2020, to May 11, 2020, separates into Δt=24h  accumulative slices (shown on the right) and Δt=4h  temporal slices (shown on the left). Both charts clearly demonstrate a phase transition between slices 360 to 390 and slices 61 to 66, respectively. Additionally, potential predictors emerge between slices 270 and 280 in the left chart. The shaded “Critical Area” corresponds to the initial period of reported arson attacks and real-world consequences.

We use the term ’phase transition’ heuristically to describe observed shifts in misinformation spread. Instead of the occupation probability, which parameterizes the phase transition in percolation phenomena, here we just have the time parameter. In some sense, we could think of a qualitative comparison with self-organized criticality phenomena [53]. In this exploratory context, we identify empirical patterns, such as the rapid tweet volume increase from April 1 to April 6, 2020 (slices 360–390, Fig 4), as a “critical shift” from sporadic to global propagation, raising awareness of tipping points in DWs.

Qualitatively, a phase transition can be investigated by identifying a parameter whose changes can drive the systems from one phase to another and by keeping track of some observable which characterizes the phase. In the case of liquid-to-gas transition, the parameter is, of course, temperature, and the observable is the density of water. In the case of transition to percolation, the parameter is a sort of probability that pairs of individuals have in establishing one contact, and the observable is the size of the largest group of individuals (cluster). In this section, we argue that the concept of phase transitions is integral to our understanding of DW. In particular, we use it to describe the critical moment when the propagation of misinformation abruptly accelerates, shifting from a slow and steady pace to a rapid, wildfire-like spread. At the same time, the phase transition quantitatively derives the three phases of the DW under investigation; see our discussions of Fig 4.

The 5G COVID-19 DW we analyze in this paper exhibits patterns suggestive of phase transition-like characteristics in its spread and contact pattern, indicating that this metaphor might apply to other DWs as well. The left side of Fig 4 presents temporal slices, specifically time slices containing only temporary interaction networks for the period from February 1, 2020, to May 11, 2020. These slices capture both the number of contacts as described in Equation 1 and the visualization of different user counts. Further, a slice (on the left) encompasses the interaction network composed of tweets, retweets, comments, and quotes in time intervals of four hours each. The number of contacts and different users reveals patterns suggestive of a shift between slices 360 and 390, corresponding to the period from April 1 to April 6, 2020. This period precisely precedes and follows the first arson attacks. These observations make it plausible to argue that the categorization of DWs into three phases, namely before, during, and after real-world consequences, as qualitatively proposed by Langguth et al. [15], can also be assessed quantitatively.

**Fig 5:**
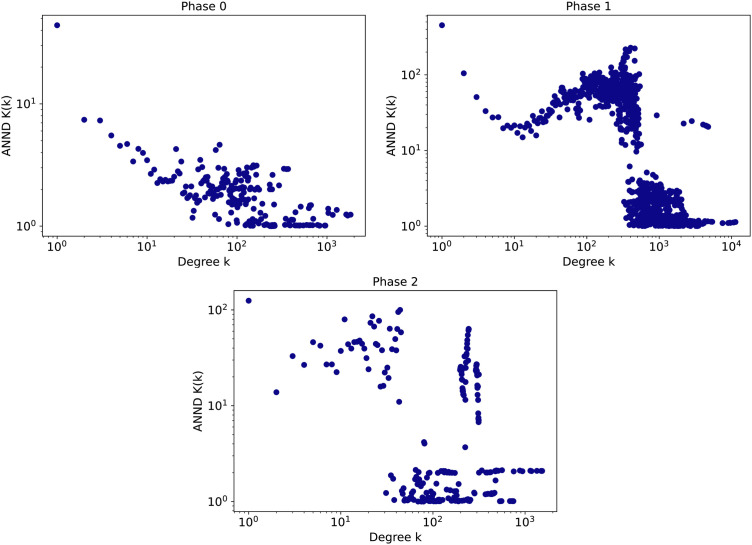
The average nearest neighbour degree (ANND) function at three different stages: (Left) before the phase transition (before slice 360), (Middle) during the phase transition (between slices 360 and 390) and (Right) after phase transition (after slice 390).

Fig 5 shows further evidence that the three stages of a phase transition in time occur during the 5G COVID-19 DW. While overall, all three phases appear dominantly disassortative, the average nearest neighbor degree, as well as its variance, significantly vary among these phases. Moreover, Fig 5 shows the average nearest neighbor degree dependence on the node degree in a qualitatively different way. Before and after the transition, there is a clear degree-range separation: for $\begin{array}{c} k≲400\; \end{array}$ the nearest neighbor degree is typically larger than for $\begin{array}{c} k≳400\; \end{array}$. At the transition, the distribution of the nearest neighbor degree seems to closely follow a power-law. This variance could represent a fruitful area for future research and a potential unique feature of DWs.

### Confluence of coalescing narratives

In the study of community dynamics over time, it is crucial to recognize that a community, in this sense, represents more than just a group of users interconnected through high volumes of interaction. In our network, an edge represents an undirected contact, indicating a larger discourse occurring within a particular timeframe. We refer to this discourse as a narrative. This terminology draws from the findings of Langguth et al. [15], who suggest that DWs, particularly the 5G COVID-19 DW, initially combine followers from various conspiracy narratives. There wasn’t one specific source for the DW. Instead, various conspiracy theorist groups had already been discussing anti-vaccination and anti-5G ideas prior to the COVID-19 pandemic, which then provided an opportunity for these narratives to merge.

**Fig 6:**
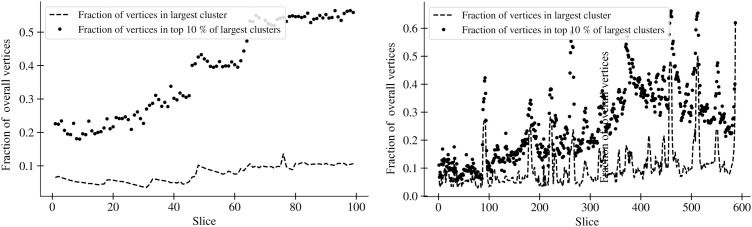
Evolution of user clustering in the analyzed DW from February 1, 2020, to May 10, 2020, represented in time slices on the x-axis. The left subplot illustrates the situation with accumulative slices and a Δt=24h . It shows a clear increase in the relative size of the top 10% of all clusters. This implies a growing participation in the 5G-COVID conspiracy narrative, with more users becoming part of clusters other than the largest cluster. The subplot on the right, displaying temporal slices with a Δt=4h , shows a similar trend. It is noteworthy that the identity of the largest cluster changes over time, i.e., the largest cluster at a given time does not necessarily contain the same users as the largest cluster at a later time.

Fig 6 portrays the evolution of user communities in the analyzed DW from February 1, 2020, to May 10, 2020. This is represented as time slices on the *x*-axis. The left subplot, indicating accumulative slices and a Δt=24h , shows a clear increase in the relative size of the 10% of the largest communities. However, the proportion of users belonging to the largest community does not increase as significantly. The subplot on the right, which represents temporal slices with a Δt=4h , shows a similar trend. Importantly, the composition of the largest community changes over time, meaning the largest community at a given moment may not include the same users as the largest community at a later moment.

When comparing the growth in relative size of the largest community with that of the top 10% of all communities, we observe that the latter grows at a slower rate. This suggests increasing participation in the 5G-COVID conspiracy narrative, with a growing number of users becoming part of the largest community over time. Therefore, a trend emerges toward the merging of different narratives.

### Influential users appear associated with amplifying large-scale conversations

A critical aspect of the dynamic evolution of DWs is the role of influential users. These users, often characterized by a high degree centrality, appear associated with shape the course of large-scale conversations. Given the network structure of social media platforms like Twitter, a message from an influential user can quickly reach a vast audience, potentially altering the trajectory of an ongoing narrative or sparking a new one.

**Fig 7:**
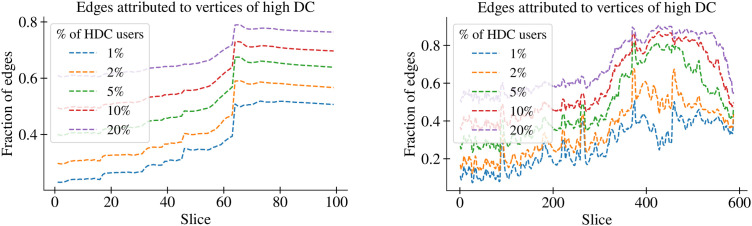
Correlation between total contacts and the fraction attributable to the most active users, gauged by degree centrality. The left subplot reveals the percentage of vertices in relation to the total number in an accumulative slice with a Δt=24h  interval, marking the transition around slice 63. The changing ratio of connections during this transition suggests that highly active users interact with a larger set of distinct users compared to those with lower activity levels. Furthermore, the figure indicates a potential predictor around slice 45, which appears to be primarily noticeable to the active users. The right hand plot presents the same relationship for the temporal slices with Δt=4h . Here, we observe similar patterns as on the left. Additionally, the gap becomes apparent between the most active 2% of users and the most active 5%, 10%, or 20% between slices 350 and 470, underlining the influential role of a small proportion of highly active users in shaping the network dynamics. The timeframe of the “Critical Area” (arson attacks and real-world consequences) aligns with the transition observed around slice 63 (left) and slices 360-390 (right).

Fig 7 illustrates the correlation between total contacts and the fraction attributable to the most active users, gauged by degree centrality. The left subplot reveals the percentage of vertices in relation to the total number in an accumulative slice with a Δt=24h  interval, marking the transition around slice 63. The changing ratio of connections during this transition suggests that highly active users interact with a larger set of distinct users compared to those with lower activity levels. The changing ratio of connections during this transition suggests that highly active users interact with a larger set of distinct users compared to those with lower activity levels. Furthermore, the figure indicates a potential predictor around slice 45, which appears to be primarily noticeable to the active users.

The right-hand plot presents the same relationship for the temporal slices with Δt=4h . Here, we observe similar patterns as on the left. Additionally, the gap becomes apparent between the most active 2% of users and the most active 5%, 10%, or 20% between slices 350 and 470, underlining the influential role of a small proportion of highly active users in shaping the network dynamics. Our analysis of the 5G-COVID-19 DW reveals that these influential users appear associated with the formation and growth of the largest community, as well as with the overall narrative evolution within the DW, though causality remains exploratory and could be tested via simulations or counterfactual network analyses. Notably, users with a high degree centrality are correlated with greater reach in the narrative dynamics compared to those with lower activity levels. Furthermore, our data suggests that influential users may act as connectors between different narratives, potentially contributing to the growth of the DW. This finding is consistent with the idea that the confluence of coalescing narratives is a fundamental characteristic of DWs.

In the context of mitigating the impact of harmful DWs, our findings underscore the importance of monitoring the activity of influential users. Effective strategies could include promoting accurate information through these users or mitigating their influence if they are spreading harmful narratives. Our analysis, however, also highlights the complexity of this task. As the identity of the largest community is not constant over time, so too are the influential users within it. This fluidity necessitates dynamic strategies for monitoring and intervention, tuned to the temporal evolution of the DW.

### Examination of pre-transition phenomena and exploration of potential predictors

In the realm of DWs, understanding the dynamics prior to a transition phase can yield critical insights into the potential predictors of such large-scale shifts. Our research illuminates several pre-transition phenomena that suggest impending changes in the narrative or the community structure. We observe a small spike visible around slice 260 in Fig 4 (left), which portrays the time or time slice on the x-axis and the number of users and user contacts on the y-axis. This peak, preceding the actual transition, could indicate that a topic is gaining potential to generate wider reach, serving as a visible spark that signals an upcoming phase shift.

Before the transition, we also observe modifications in user behavior patterns. Specifically, the most active users begin to expand their range of contacts. These users, who typically have a high degree centrality, start interacting with a more extensive set of unique users compared to less active ones. This observation reinforces the central role of influential users in guiding the DW into a new phase, a notion we discussed in the previous section.

We also see changes in the structural properties of the network before the transition. For instance, the ratio of connections, i.e., the number of communications with different actors, shifts during the transition. This suggests the possibility of using network structure changes as predictive indicators of a DW phase transition. These observations suggest that, with further study and refinement, we might be able to establish a predictive model for DW transitions. Such a model would not only enhance our understanding of DW dynamics but could also provide actionable insights to contain or guide these narratives. Consequently, our future research will focus on refining these potential predictors and testing their predictive power across different contexts.

## Discussion and conclusion

In this paper, we address the dynamics and temporal evolution of the COVID-19 and 5G DW from early 2020. By processing a massive-scale Twitter dataset, we trace the course of this DW beyond the investigation of individual information spreading cascades. Rather, we examine the underlying communication in interaction networks and thus provide a more holistic view of the dynamics that underlie this DW. We observe that both the average count of user contacts and the average community sizes over the total duration of the DW exhibit visual patterns suggestive of power-law distributions. This exploratory evidence points to structural similarities with other communication networks, while formal statistical validation remains outside the scope of this study.

Our findings also connect to cascade models in networks, particularly the distinction between local and global cascades. Tightly knit communities in our DW resemble *local cascades*, where information primarily circulates within small, cohesive groups, whereas the rapid, cross–community spread we observe during the critical phase aligns with a *global cascade*. Framing the observed phase transition–like dynamics in terms of cascades offers a bridge between our complex-systems perspective on DWs and established contagion theory, and helps explain how activity can tip from contained discussion to platform-wide propagation [13,54].

Based on the study of these dynamics, we propose a framework that not only allows for accessing the three stages of this DW quantitatively but also allows for the application of well-established methods to understand this DW through the lens of physics. Physicists are able to predict phase transitions by identifying drivers. Temperature, for example, is the driver for the transition water undergoes between gas, liquid and solid phases. In this work, we identify potential candidates for drivers in what appears to be a phase transition of a DWs, based on the underlying communication, thereby paving the way towards a potential DW prediction model. However, formally establishing that these dynamics constitute a true phase transition remains to be shown, and further work is required to demonstrate that such drivers apply to other DWs.

Thus, we observe a confluence of coalescing narratives by studying the community dynamics over the course of this DW. We ask the reader to recall that a community in a time slice of a temporal interaction network is a group of people, in our case, Twitter users, talking to each other (about a topic) within a certain time window.

Furthermore, we identify a subset of all users involved in this DW that could be a major driving force for the transition to a global event. This result suggests that potential drivers can be found in the underlying characteristics of the communication of this user group. As with coalescing narratives, further studies need to show that this result also manifests in other DWs.

Our study’s findings have considerable real-world implications. Unraveling these dynamics can be a potent tool for entities wishing to manage or control information propagation. For democratic societies, these tools can be invaluable in identifying and mitigating nascent extremist groups or disinformation campaigns. On the other hand, they could potentially be exploited to suppress democratic dissent in totalitarian regimes. Given the high stakes, an effective response to DWs requires an approach that harmonizes technological tools with education, media literacy, and an informed public. Moreover, it is critical to ensure democratic accountability for those who wield these powerful tools.

To conclude, this study represents an effort to understand the dynamics of a DW within complex temporal interaction networks, with results shedding new light on the societal-scale understanding of these phenomena. Our ultimate objective is to devise methods that can predict and mitigate the spread of harmful misinformation.

## Limitations

This study focuses solely on the 5G-COVID DW, limiting its findings to this single case. The observed patterns, such as phase transition-like behavior and the role of influential users, may not hold across the broader DW ecosystem and require further validation. Comparative analysis with other misinformation events (e.g., election fraud claims or vaccine conspiracy narratives) is essential to assess generalizability.

Furthermore, our network analysis treats all nodes as users and does not explicitly distinguish between human actors and automated accounts (bots) or organizational entities. It is possible that a portion of the high-degree “influential” nodes identified in our study are automated amplification accounts. While distinguishing these is beyond the scope of this exploratory study, future work should consider how bot activity contributes to the observations.

Moreover, we deliberately adopted a conceptual and exploratory approach, inspired by social science methodologies. Rather than presenting strict statistical physics analogies, we frame the “phase transition” concept as a metaphorical lens to highlight sudden shifts in misinformation spread. This shift addresses concerns about methodological rigor and statistical robustness by focusing on qualitative patterns, descriptive analyses, and interdisciplinary implications. While our extensive dataset and novel use of temporal interaction networks and community dynamics provide a valuable framework for understanding DWs, formally demonstrating that these dynamics constitute a physical phase transition remains outside the scope of this study. The intention is to raise awareness of phase transition–like behaviors in social media dynamics and to encourage future empirical and comparative work.

### Future work

Building on this study, future research should explore multiple DWs to validate the identified patterns and drivers. Comparative studies across diverse cases, such as health-related misinformation or political propaganda, could test the framework‘s applicability and refine prediction models. Multi-platform data integration (e.g., Twitter, YouTube, Facebook) would also enhance understanding of cross-channel dynamics.

Furthermore, future studies should also compare different centrality measures, such as eigenvector, betweenness, and hub/authority metrics, and examine whether distinguishing edge types (e.g., retweets vs. replies) alters the observed dynamics.

In addition, we plan to investigate the mechanisms underlying the confluence of narratives, and assess whether this process can be modeled as a building block of a driver. We encourage future work to validate and expand upon the identified patterns and hypotheses, while emphasizing that single-case insights require cross-validation.

Finally, interdisciplinary collaboration and applied testing—such as developing early-warning tools for policymakers and journalists—will be essential to translate these conceptual insights into practical strategies for mitigating harmful DWs.

## References

[1] A Brief History of the Internet by the Board of Regents of the University System of Georgia. Available from: https://www.usg.edu/galileo/skills/unit07/internet07_02.phtml

[2] Peattie S. The Internet as a Medium for Communicating with Teenagers. Soc Market Quart. 2007;13(2):21–46. doi: 10.1080/15245000701326343

[3] boyd danah m., Ellison NB. Social Network Sites: Definition, History, and Scholarship. J Comput-Mediat Commun. 2007;13(1):210–30. doi: 10.1111/j.1083-6101.2007.00393.x

[4] Network IFC. IFCN‘s code of principles transparency report for 2020. Report.

[5] Clayton K, Blair S, Busam JA, Forstner S, Glance J, Green G, et al. Real Solutions for Fake News? Measuring the Effectiveness of General Warnings and Fact-Check Tags in Reducing Belief in False Stories on Social Media. Polit Behav. 2019;42(4):1073–95. doi: 10.1007/s11109-019-09533-0

[6] Data R. Put More of Your Business Data to Work—From Edge to Cloud. A Seagate Technology Report-2020. 2020.

[7] Schroeder DT, Cha M, Baronchelli A, Bostrom N, Christakis NA, Garcia D, et al. How Malicious AI Swarms Can Threaten Democracy. 2025. Available from: https://arxiv.org/abs/2506.06299 10.1126/science.adz1697 41570131

[8] Banaji S, Bhat R, Agarwal A, Passanha N, Sadhana PM. WhatsApp vigilantes: An exploration of citizen reception and circulation of WhatsApp misinformation linked to mob violence in India. London School of Economics and Political Science; 2019.

[9] Twickel Nv. Fake twitter account pumps up oil prices. 2012. Available from: https://www.themoscowtimes.com/2012/08/07/fake-twitter-account-pumps-up-oil-prices-a16852

[10] Biswas S. Social Media and the India Exodus. 2012. Available from: https://www.bbc.com/news/world-asia-india-19292572

[11] Fisher M, Cox JW, Hermann P. Pizzagate: From rumor, to hashtag, to gunfire in DC. Washington Post. 2016;6:8410–5.

[12] Howell L. Digital wildfires in a hyperconnected world. 2013. Available from: http://reports.weforum.org/global-risks-2013/risk-case-1/digital-wildfires-in-a-hyperconnected-world/

[13] Watts DJ. A simple model of global cascades on random networks. Proc Natl Acad Sci U S A. 2002;99(9):5766–71. doi: 10.1073/pnas.082090499 16578874 PMC122850

[14] Christensen K, Moloney NR. Complexity and criticality. Imperial College University Press; 2005.

[15] Langguth J, Filkuková P, Brenner S, Schroeder DT, Pogorelov K. COVID-19 and 5G conspiracy theories: long term observation of a digital wildfire. Int J Data Sci Anal. 2023;15(3):329–46. doi: 10.1007/s41060-022-00322-3 35669096 PMC9137448

[16] Chen Y, Conroy NJ, Rubin VL. Misleading online content: recognizing clickbait as “false news”. In: Proceedings of the 2015 ACM on workshop on multimodal deception detection. 2015. p. 15–9.

[17] Gupta A, Lamba H, Kumaraguru P, Joshi A. Faking sandy: characterizing and identifying fake images on twitter during hurricane sandy. In: Proceedings of the 22nd international conference on World Wide Web. 2013. p. 729–36.

[18] Castillo C, Mendoza M, Poblete B. Information credibility on twitter. In: Proceedings of the 20th international conference on World wide web. 2011. p. 675–84. doi: 10.1145/1963405.1963500

[19] Ruchansky N, Seo S, Liu Y. Csi: A hybrid deep model for fake news detection. In: Proceedings of the 2017 ACM on Conference on Information and Knowledge Management. 2017. p. 797–806.

[20] Jin Z, Cao J, Zhang Y, Luo J. News Verification by Exploiting Conflicting Social Viewpoints in Microblogs. AAAI. 2016;30(1). doi: 10.1609/aaai.v30i1.10382

[21] Pogorelov K, Schroeder DT, Burchard L, Moe J, Brenner S, Filkukova P, et al. FakeNews: Corona Virus and 5G Conspiracy Task at MediaEval 2020. In: MediaEval; 2020.

[22] Pogorelov K, Schroeder DT, Brenner S, Maulana A, Langguth J. Combining tweets and connections graph for fakenews detection at mediaeval 2022. In: Multimedia Benchmark Workshop; 2022.

[23] Schroeder DT, Schaal F, Filkukova P, Pogorelov K, Langguth J. WICO Graph: a Labeled Dataset of Twitter Subgraphs based on Conspiracy Theory and 5G-Corona Misinformation Tweets. 2021.

[24] Vosoughi S, Roy D, Aral S. The spread of true and false news online. Science. 2018;359(6380):1146–51. doi: 10.1126/science.aap9559 29590045

[25] Newman MEJ, Girvan M. Finding and evaluating community structure in networks. Phys Rev E Stat Nonlin Soft Matter Phys. 2004;69(2 Pt 2):026113. doi: 10.1103/PhysRevE.69.026113 14995526

[26] Blondel VD, Guillaume J-L, Lambiotte R, Lefebvre E. Fast unfolding of communities in large networks. J Stat Mech. 2008;2008(10):P10008. doi: 10.1088/1742-5468/2008/10/p10008

[27] Traag VA, Waltman L, van Eck NJ. From Louvain to Leiden: guaranteeing well-connected communities. Sci Rep. 2019;9(1):5233. doi: 10.1038/s41598-019-41695-z 30914743 PMC6435756

[28] Starbird K. Examining the Alternative Media Ecosystem Through the Production of Alternative Narratives of Mass Shooting Events on Twitter. ICWSM. 2017;11(1):230–9. doi: 10.1609/icwsm.v11i1.14878

[29] Del Vicario M, Bessi A, Zollo F, Petroni F, Scala A, Caldarelli G, et al. The spreading of misinformation online. Proc Natl Acad Sci U S A. 2016;113(3):554–9. doi: 10.1073/pnas.1517441113 26729863 PMC4725489

[30] Friggeri A, Adamic L, Eckles D, Cheng J. Rumor Cascades. ICWSM. 2014;8(1):101–10. doi: 10.1609/icwsm.v8i1.14559

[31] Kelion L. Coronavirus: 20 suspected phone mast attacks over Easter. 2020. Available from: https://www.bbc.com/news/technology-52281315

[32] Staff R. False claim: Video shows a 5g mast burning in Nigeria. 2020. Available from: https://www.reuters.com/article/uk-factcheck-5g-nigeria-burning-idUSKBN22225W

[33] Lamoureux M. It looks like the 5G conspiracy theory fires have arrived in Canada. 2020. Available from: https://www.vice.com/en/article/g5px8x/5g-conspiracy-theory-coronavirus-cell-tower-fires-canada

[34] Pogorelov K, Schroeder DT, Filkuková P, Brenner S, Langguth J. Wico text: a labeled dataset of conspiracy theory and 5g-corona misinformation tweets. In: Proceedings of the 2021 Workshop on Open Challenges in Online Social Networks. 2021. p. 21–5.

[35] Collins K. Violence, arson, abuse: The real-world consequences of those false 5G conspiracies. 2020. Available from: https://www.cnet.com/tech/services-and-software/fake-5g-coronavirus-theories-have-real-world-consequences/

[36] Gilbert P. Vodacom, MTN towers burnt in SA by alleged 5G conspiracy theorists. 2021. Available from: http://www.connectingafrica.com/author.asp?section_id=761doc_id=766499

[37] Staff N. Police say cell phone tower fire in Scarborough considered arson; 2021. Available from: https://toronto.citynews.ca/2021/03/31/fire-crews-battle-burning-cell-phone-tower-in-scarborough/

[38] Stanley HE. Introduction to Phase Transitions and Critical Phenomena. Oxford University Press; 1987.

[39] Schroeder DT, Langguth J, Burchard L, Pogorelov K, Lind PG. The connectivity network underlying the German’s Twittersphere: a testbed for investigating information spreading phenomena. Sci Rep. 2022;12(1):4085. doi: 10.1038/s41598-022-07961-3 35260708 PMC8902855

[40] Schroeder DT, Pogorelov K, Langguth J. FACT: a Framework for Analysis and Capture of Twitter Graphs. In: 2019 Sixth International Conference on Social Networks Analysis, Management and Security (SNAMS). 2019. p. 134–41. doi: 10.1109/snams.2019.8931870

[41] Burchard L, Schroeder DT, Becker S, Langguth J. Resource Efficient Algorithms for Message Sampling in Online Social Networks. In: 2020 Seventh International Conference on Social Networks Analysis, Management and Security (SNAMS). 2020. p. 1–8. doi: 10.1109/snams52053.2020.9336530

[42] Barabasi A, Albert R. Emergence of scaling in random networks. Science. 1999;286(5439):509–12. doi: 10.1126/science.286.5439.509 10521342

[43] Fortunato S. Community detection in graphs. Physics Reports. 2010;486(3–5):75–174. doi: 10.1016/j.physrep.2009.11.002

[44] Faloutsos M, Faloutsos P, Faloutsos C. On power-law relationships of the Internet topology. SIGCOMM Comput Commun Rev. 1999;29(4):251–62. doi: 10.1145/316194.316229

[45] Adamic LA, Huberman BA. Power-Law Distribution of the World Wide Web. Science. 2000;287(5461):2115–2115. doi: 10.1126/science.287.5461.2115a

[46] Jeong H, Tombor B, Albert R, Oltvai ZN, Barabási AL. The large-scale organization of metabolic networks. Nature. 2000;407(6804):651–4. doi: 10.1038/35036627 11034217

[47] Li L, Alderson D, Doyle JC, Willinger W. Towards a Theory of Scale-Free Graphs: Definition, Properties, and Implications. Internet Mathematics. 2005;2(4):431–523. doi: 10.1080/15427951.2005.10129111

[48] Clauset A, Shalizi CR, Newman MEJ. Power-Law Distributions in Empirical Data. SIAM Rev. 2009;51(4):661–703. doi: 10.1137/070710111

[49] Mislove A, Marcon M, Gummadi KP, Druschel P, Bhattacharjee B. Measurement and analysis of online social networks. In: Proceedings of the 7th ACM SIGCOMM conference on Internet measurement. 2007. p. 29–42. doi: 10.1145/1298306.1298311

[50] Albert R, Barabási A-L. Statistical mechanics of complex networks. Rev Mod Phys. 2002;74(1):47–97. doi: 10.1103/revmodphys.74.47

[51] Sugiyama Y, Fukui M, Kikuchi M, Hasebe K, Nakayama A, Nishinari K, et al. Traffic jams without bottlenecks—experimental evidence for the physical mechanism of the formation of a jam. New J Phys. 2008;10(3):033001. doi: 10.1088/1367-2630/10/3/033001

[52] Moussaïd M, Kämmer JE, Analytis PP, Neth H. Social influence and the collective dynamics of opinion formation. PLoS One. 2013;8(11):e78433. doi: 10.1371/journal.pone.0078433 24223805 PMC3818331

[53] Christensen K, Moloney NR. Complexity and criticality. vol. 1. Imperial College Press; 2005.

[54] Watts DJ, Dodds PS. Influentials, networks, and public opinion formation. J Consum Res. 2007;34(4):441–58.

